# Transcriptional changes related to secondary wall formation in xylem of transgenic lines of tobacco altered for lignin or xylan content which show improved saccharification

**DOI:** 10.1016/j.phytochem.2011.10.009

**Published:** 2012-02

**Authors:** Charis M. Cook, Arsalan Daudi, David J. Millar, Laurence V. Bindschedler, Safina Khan, G. Paul Bolwell, Alessandra Devoto

**Affiliations:** a School of Biological Sciences, Royal Holloway, University of London, Egham, Surrey TW20 0EX, UK; b Department of Medicine, Royal Free & University College Medical School, University College London, Rowland Hill Street, London NW3 2PF, UK; c Department of chemistry, University of Reading, Whiteknights, Reading RG6 6AS, UK

**Keywords:** ADH, bifunctional alcohol/UDP glucose dehydrogenase, AIM, acetone-insoluble material, CESA3, cellulose synthase, CSLD, cellulose synthase-like D, PAL, phenylalanine ammonia lyase, C4H, cinnamate 4-hydroxylase, C3H, coumaroyl-ester-3-hydroxylase, COMT, caffeic acid *O*-methyl transferase, CCOMT, caffeoyl-CoA methyl-transferase, CCR, cinnamoyl-CoA reductase, CAD, cinnamyl alcohol dehydrogenase, HQT, hydroxycinnamoyl-CoA:quinate hydroxycinnamoyltransferase, SUSY, sucrose synthase, UGD, UDP-glucose dehydrogenase, UXS, UDP-glucuronate decarboxylase, Tobacco, *Nicotiana tabacum*, Solanaceae, Cell wall, Lignin, Xylan, Antisense, Saccharification

## Abstract

In this study, an EST library (EH663598–EH666265) obtained from xylogenic tissue cultures of tobacco that had been previously generated was annotated. The library proved to be enriched in transcripts related to the synthesis and modification of secondary cell walls. The xylem-specific transcripts for most of the genes of the lignification and xylan pathways were identified and several full-length sequences obtained. Gene expression was determined in available tobacco lines down-regulated for enzymes of the phenylpropanoid pathway: *CINNAMATE 4-HYDROXYLASE* (*sc4h*), *CINNAMOYL-COA REDUCTASE* (*asccr*) and lignification-specific peroxidase (*asprx*). In addition, lines down-regulated in the nucleotide-sugar pathway to xylan formation through antisense expression of *UDP-GLUCURONIC ACID DECARBOXYLASE* (*asuxs*) were also analysed. It is shown herein that most transcripts were down-regulated for both lignin and xylan synthesis pathways in these lines, while *CELLULOSE SYNTHASE A3* was up-regulated in lignin-modified lines. The analysis indicates the existence of interdependence between lignin and xylan pathways at the transcriptional level and also shows that levels of cellulose, xylan and lignin are not necessarily directly correlated to differences in transcription of the genes involved upstream, as shown by cell wall fractionation and sugar analysis. It is therefore suggested that cell wall biosynthesis regulation occurs at different levels, and not merely at the transcriptional level. In addition, all lines analyzed showed improved enzymic saccharification of secondary but not primary walls. Nevertheless, this demonstrates potential industrial applicability for the approach undertaken to improve biomass utility.

## Graphical abstract

**Graphical abstract:**

An EST library derived from xylogenic cells has been used to direct transcriptional profiling of genetically engineered tobacco lines which show improved biomass saccharification.

**Figure:**
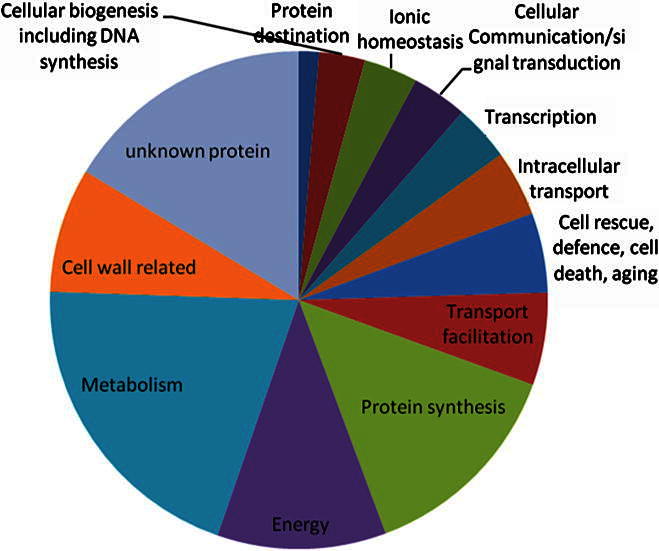

**Highlights:**

► Description of a xylogenic EST. ► Cell wall consequences of down-regulation of lignin and xylan. ► Improved saccharification of secondary walls but not primary walls. ► Transcriptional analysis of cell wall biosynthesis genes in modified transgenic lines. ► Identification of transcription factors.

## 1 Introduction

The efficiency of extraction and recovery of cellulose from cell walls influences many industrial processes including fibre production, pulp and paper-making and utilisation of biomass for biofuel. The cell wall is a complex laminate structure, which can be classified in dicots into three distinct zones, the middle lamella, the primary wall and the secondary wall. The middle lamella is shared by two contiguous cells. The primary cell wall is composed chiefly of interwoven domains, of which the cellulose–xyloglucan framework is the main contributor of the biomass. However the biomass with the highest industrial applicability is mainly derived from secondary walls, which consist of three distinct layers (S1, S2, S3), easily distinguishable at an ultrastructural level from differences in the orientation of their cellulose microfibrils. The transition from primary to secondary cell wall synthesis is marked by the cessation of pectin deposition and a noted increase in the synthesis and deposition of cellulose, hemicelluloses and lignins. The cellulose and noncellulosic polysaccharides of the secondary cell wall are qualitatively distinct from those found in the primary cell walls. The major differences are in the hemicellulose components of the secondary cell wall, which are primarily xylans and mannans. Like in any complex composite material, the supramolecular organisation between cellulose, hemicelluloses and lignins in cell walls determines the properties of plant fibres. Although there have been many studies identifying structural genes and transcription factors involved in wood formation in dicots and gymnosperms (Sterky et al., 1998; Paux et al., 2004; Aspeborg et al., 2005; Brown et al., 2005; Pavy et al., 2005) understanding of the extent of co-regulation of assembly of these three components is still limited.

This area of study is of particular importance due to the economic value of woody tissue both as a product in its own right and as a structural component for its rheological properties. It is highly desirable to modify the woody composition of plants and several major projects have been initiated in this area internationally (Anterola and Lewis, 2002; Boudet et al., 2003; Boerjan et al., 2003). These have generated transgenic lines with reduced lignin which has realised proven benefits to pulp and paper-making. Lines modified in xylan content have also been generated and showed useful changes in cellulose extractability and quality following chemical digestibility (Bindschedler et al., 2007). Such proof of concept studies form the basis of optimism that plant biomass can be engineered successfully for many industrial processes. This has been extended recently to biofuel where microbial saccharification as a prerequisite to ethanol generation is dependent upon lignin and hemicellulose content. Lignin modification has already been shown to improve enzymatic release of sugars (Chen and Dixon, 2007; Kavousi et al., 2010).

The advances in this area using EST and genomic-based approaches are subject to limitations due to poor annotation of the databases especially with respect to cell wall biosynthesis and its regulation. To facilitate the identification of new target genes to exploit, a model system was previously developed for tracheid development in tobacco consisting of a tobacco suspension cell culture line transformed with a constitutively expressed Tcyt gene (Blee et al, 2001a). Such cultured xylogenesis systems, first developed for Zinnia (Demura et al., 2002), continue to be of great use in studying vascular differentiation. Recent novel innovations for tobacco include inducible transcription factor expression leading to almost complete xylogenesis (Yamaguchi et al., 2010). In the Tcyt-dependent system, secondary cell walls are continually being made under the influence of *in vivo* generation of cytokinin. Our approach to novel gene discovery utilised this model xylogenic tissue culture system for proteomics of the secretory system (Millar et al., 2009). An EST library generated using this system by Blee et al (2001a) is used here to direct transcriptional profiling of a panel of transgenic lines altered in lignin or hemicelluloses. These lines include: the sense orientation transgenic tobacco line for *CINNAMATE 4-HYDROXYLASE* (referred to in the text as *sc4h*), altered for the flux into the lignin pathway (Blee et al., 2001); the antisense transgenic lines for the *CINNAMOYL-COA REDUCTASE* (*asccr*), altered for monolignol composition (Piquemal et al., 1998); the lignification specific peroxidase *TOBACCO PEROXIDASE 60* (*asprx*), which was modified for lignin polymerisation (Blee et al., 2003; Kavousi et al., 2010) and the *UDP-GLUCURONATE* DECARBOXYLASE (*asuxs*), modified for flux into the xylan synthesis pathway (Bindschedler et al., 2007). Furthermore, all these lines showed improved enzymic saccharification demonstrating potential industrial applicability.

## 2 Results

### 2.1 EST library characterisation and analysis of custom expression array

**Fig. 1:**
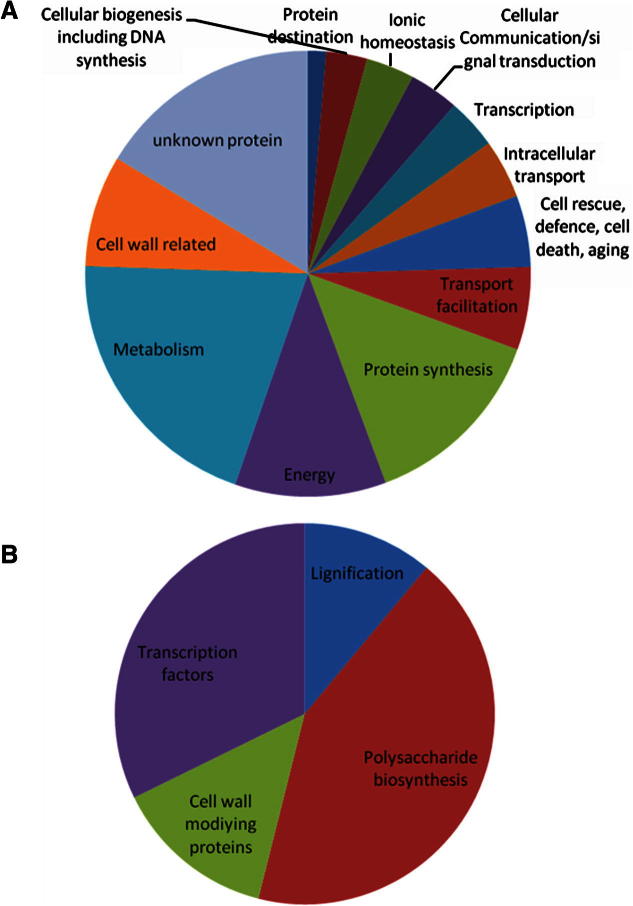
Distribution of ESTs among categories. (A) Distribution amongst all standard categories. The selection of ESTs represented totals 2147 and excludes those with no significant hit on NCBI and those whose best alignment was to a completely unannotated nucleotide sequence; and (B) distribution amongst cell wall and regulatory transcription factors.

A collection of ESTs was obtained from a xylogenic tobacco cell culture that was characterized previously (Blee et al., 2001b). Out of a total of 2976 cDNA clones subjected to a single pass sequence from the 5′ end, 2668 sequences were obtained and deposited in Genbank (EH663598–EH666265). These have been updated and as of 04/2010 the annotations against highest match and nearest Arabidopsis paralogue are shown in Supplemental data I. Initial characterisation of those clones yielding sequence data suggests that 225 (13%) are related to cell wall functions which is comparable to the average percentage often found for cell wall related sequences in plant genomes and ESTs (9%). No sequence similarity was found for 357 (13.4%) ESTs towards any proteins in the public databases. A further 313 (16% of the whole database) clones were classified as unknown proteins (Fig. 1) and 185 (7%) were significantly similar to an unannotated sequence. Therefore, there is considerable capacity for the discovery of novel genes involved in cell wall biosynthesis and modification. The remaining 1608 (51.5%) ESTs significantly matched sequences with assigned functions. A subset of ESTs clearly involved in cell wall biosynthesis and modification is summarised in Table 1. The overall functional distribution is shown in Fig. 1.

The results obtained from microarray transcription profiling of T-cyt transformed cultures 3 days after subculture are also shown in Supplementary data I. The transcripts have been selected because they deviate by more than 2-fold expression. Up-regulated genes include those typical of secondary cell-wall forming tissues, *S-ADENOSYL METHIONINE* (*SAM*) *SYNTHASE*, *COPPER OXIDASE*, *GLYCINE-RICH PROTEIN*, *POLYGALACTURONASE*, *β-1*,*3 GLUCANASE*, *ALCOHOL DEHYDROGENASE* with UDP-glucose dehydrogenase activity and a number of glycosyl transferases. The down-regulated genes include *BAX INHIBITOR PROTEIN* in cell death regulation and *ENDO-XYLOGLUCAN TRANSGLYCOSYLASE* in primary wall modification. This analysis shows potential candidates for further functional analysis which goes beyond the scope of this work.

### 2.2 Gene expression and cell wall formation

Cellulose is synthesised using UDP-glucose. One possible source of UDP-glucose is through *SUCROSE SYNTHASE* (SuSy; Haigler et al., 2001) and there are six *SUSY* (EH664527, EH664745, EH664820, EH666077, EH666169, EH666255), and three invertases (EH664106, EH664540 EH665657) ESTs present in our list (Supplementary data I, Table 1). If abundance is a true reflection of elevated gene expression, this is probably related to the increased production of cellulose during secondary wall formation. Alternatively, UDP-glucose may be derived from starch as starch grains disappear rapidly in these tissues during secondary wall formation (Bolwell, 1993). Transcripts were present for amylase (EH663749), *ADP-GLUCOSE PYROPHOSPHORYLASE* (EH664505 and EH664848), and *UDP-GLUCOSE PYROPHOSPHORYLASE* (EH664780, EH664052, EH664554, EH664604, EH664969, EH665027, EH665911, EH666132, EH663806). With respect to cellulose synthesis (Persson et al., 2005), the suggested specificity with respect to this large gene family is also apparent from the ESTs here where the three found (EH663724; EH663943, EH664994) are all tobacco homologues of Arabidopsis *CELLULOSE SYNTHASE* *ATCESA3* which is associated with secondary wall synthesis and the source of the *irregular xylem* (*irx*1) mutant (Brown et al., 2005). In other systems, *CELLULOSE SYNTHASE-LIKE* (*CSL*) and *CELLULOSE SYNTHASE* (*CESA*) genes are usually represented less than 0.03% in ESTs (Kanwarpal Dhugga, Pioneer Hibred International, IA, USA personal communication), so that the low abundance seen may be consistent between examples from various species. *CESA3* was expressed in xylem tissue of wild type as described below.

Genes specific for the lignification pathway leading to monolignol synthesis are present in our list. Surprisingly, no ESTs coding for *PHENYLALANINE AMMONIA-LYASE* (*PAL*) appeared, however two different class I *CINNAMATE 4-HYDROXYLASES* (*C4H*) were found (EH664914, EH665327) which probably represent the two isoforms in the allotetraploid, *Nicotiana tabacum*. Class I forms have not been reported previously, yet the two sequences coding for full length class II forms are present by searching Genbank EH663728 is annotated as the *CYP98* coding for the next hydroxylation step in the pathway after cinnamate 4-hydroxylation, *COUMAROYL-ESTER-3-HYDROXYLASE* (*C3H*; Abdulrazzak et al., 2006; Ehlting et al., 2006). Representatives of the rest of the pathway, *CAFFEOYL-COA METHYL-TRANSFERASE* (*CCOMT*; EH665253, EH665876), *CAFFEIC ACID O-METHYL TRANSFERASE* (*COMT*; EH663855, EH665400, EH665510, EH666153), *CINNAMOYL-COA REDUCTASE* (*CCR*; EH664240, EH664699, EH666151, EH666260), *CINNAMYL ALCOHOL DEHYDROGENASE* (*CAD*; EH664137, EH664150, EH664162, EH664196, EH664225, EH664374, EH664909) and *HYDROXYCINNAMOYL-COA:QUINATE HYDROXYCINNAMOYLTRANSFERASE* (*HQT*; EH664996, EH666193) are present in our list. These include genes targeted in a number of antisense down-regulation programmes for each of these reactions (Anterola and Lewis, 2002). The only major absent EST was for *4-COUMARATE LIGASE* (*4CL*). Expression of the extant ESTs was detected by RT-PCR in xylem tissue of wild type as described below.

### 2.3 Gene expression and matrix polysaccharide formation

**Fig. 2:**
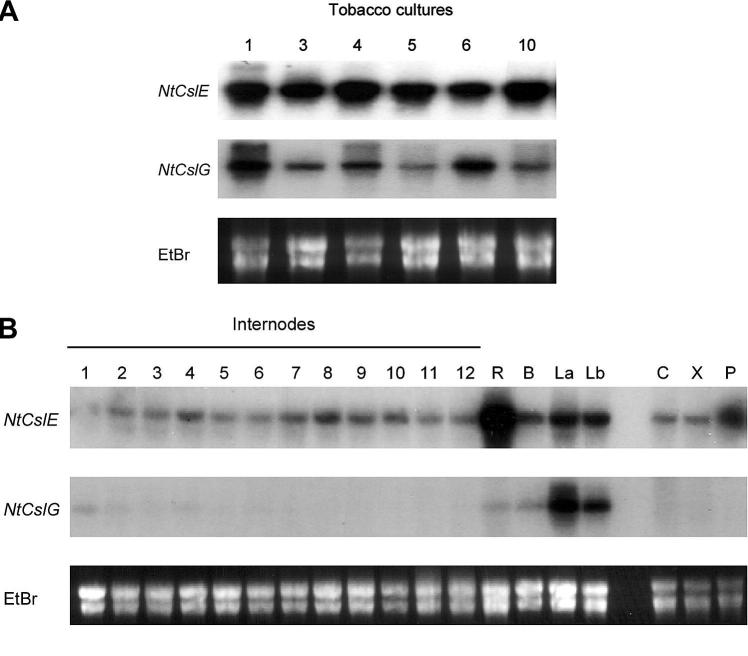
Expression analysis of CslE and CslG. Northern blots are shown for expression in (A) xylogenic tobacco cultures 1–10 days after subculture and (B) internodes 1–11, R = root, B = Buds, La = developing leaves of 0–5 cm long, Lb = older leaves of more than 10 cm long, C = cortex, P = phloem, X = xylem.

To dissect the relationship between the various components of the cell wall, it is important to understand not only the effects of the manipulation of the lignin pathway but to focus our attention also to other non-cellulosic polymers in secondary walls of dicots such as xylan. Isolation of wall proteins involved in modification and possible assembly of secondary wall xylan can also underpin future development of engineering plant fibre. For the other major target for manipulation, glucuronoarabinoxylan synthesis, the specific vascular genes required for the provision of the substrates UDP-xylose and UDP-glucuronate have also been identified (Bindschedler et al., 2005, 2007) and are all represented in our EST collection: *UDP-XYLOSE SYNTHASE* (*UXS*; EH664223), *ADH-LIKE UDP-GLUCOSE DEHYDROGENASE* (*ADH*; 20 ESTs – complete list in Supplementary data I), *UDP-GLUCOSE 6-DEHYDROGENASE* (*UGD*; EH663670), *UDP-GLUCURONATE DECARBOXYLASE* (*UXS*; EH663981, EH664621, EH664838, EH664948) and *UDP-GLUCURONATE 4-EPIMERASE* (*UG4E*; EH664555). The abundance of glycosyl transferases and polysaccharide synthases which could be identified within the ESTs are limited but *CESA3* was found to be expressed in xylem in other systems (Brown et al., 2005). In this study, two full-length *CELLULOSE SYNTHASE-LIKE* clones *CSLE* (DQ127171) and *CSLG* (DQ152918) were cloned from the Tcyt cDNA library and the steady state mRNA expression levels have been analyzed over a time course using Northern blots in both xylogenic tissue cultured cells and stems (Fig. 2). While *CSLG* mRNA levels were highest in tissues undergoing primary wall formation (Fig. 2A), C*SLE* expression was highest in tissues associated with secondary wall biosynthesis but mainly in the phloem (Fig. 2A). Another family member, *CSLD* was also found to be expressed by RT-PCR in wild type xylem tissue. However functional evidence for its role as a xylan synthase is controversial (Samuga and Joshi, 2004; Bernal et al., 2007), since other evidence from other model species underpins claims that members of the glycosyltransferase *GT43* family may be a xylan synthase (Pena et al., 2007; Brown et al., 2007). No ESTs were annotated as a *GT43* but one of the glycosyl transferases was identified as the family 47 member which is annotated as a glucuronosyl transferase originally thought to be involved in glucuronoarabinoxylan biosynthesis in Arabidopsis (Zhong et al., 2005). However, recent claims have been made for *GT47* members having xylosyl transferase activity (Brown et al., 2009; Lee et al., 2009a; Wu et al., 2009) and their down-regulation resulted in improved saccharification (Lee et al., 2009b). In previous proteomic studies, a *GT47* was detected in membranes from xylogenic tobacco cells (Millar et al., 2009).

Xylan biosynthesis is followed by assembly and possibly remodelling. Therefore, in addition to xylan synthase, secondary wall xylanase and xylan binding protein may be required. In support to this, two full length xylanase clones cDNAs (TQ152919 and DQ152919) were also obtained from our EST library.

### 2.4 Consequences of transgenesis on wall composition and saccharification efficiency of primary and secondary wall

**Fig. 3:**
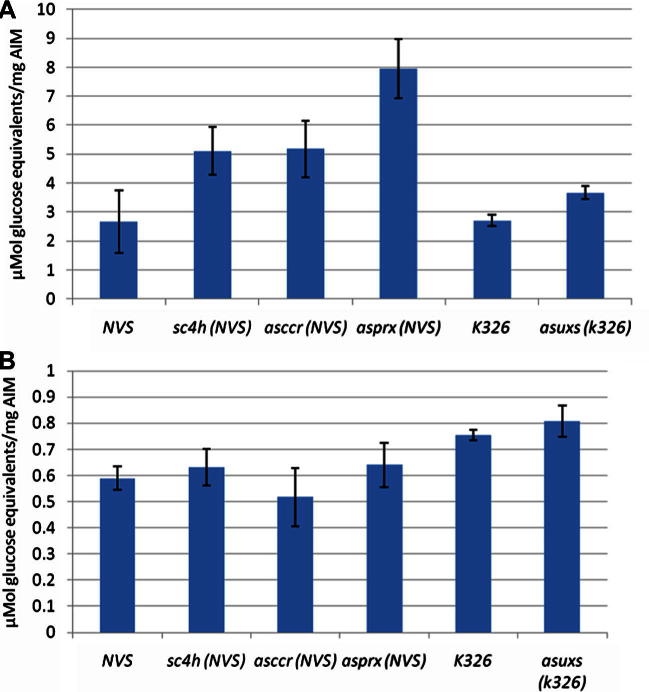
Saccharification analysis of stem (A) and leaf (B) from cell wall modified lines. (A) Sugar release from acetone insoluble material extracted from the bottom six internodes of the stems of three different plants. (B) Sugar release from acetone insoluble material extracted from the leaves of six different plants. Soluble sugar content was measured after 72 h. The data are the mean of six assays, each consisting of three separate pools of material. Bars indicate standard error.

Table 2A and B show the primary wall composition of leaf and the stem secondary cell wall composition of tobacco lines modified for lignin and xylan. Table 2A shows little change in the three main components of the primary wall between the lines confirming that transgenesis was specific to vascular walls. Changes in secondary wall composition shown in Table 2A are detailed in Table 2B (containing published and unpublished data). In addition, preliminary profiling of *asccr* lines indicated a glucose/xylose ratio of between 1.9 and 2.4, an effect which is more comparable to that seen for consequences of *CCR* manipulation in Arabidopsis, rather than in poplar (Ruel and Joseleau, personal communication). The relevant secondary cell wall composition leads to different saccharification efficiencies in the order *asuxs* < *sc4h* = *asccr* < *asprx* (Fig. 3A). The *asprx* data, at 3-fold improvement in saccharification efficiency, showed the greatest improvement and has been reported previously (Kavousi et al., 2010) but is included here for direct comparison.

Similarly, downregulation of xylan in *asuxs* improved saccharification efficiency, at 50% higher than wild-type. If translated to an industrial scale, even this improvement would be considerable. The transgenic strategy was directed towards the stem and consequently there was little change in leaf primary wall composition (Table 2A) and no improvement in saccharification (Fig. 3B).

### 2.5 Profiling of pathway genes in lines down-regulated in lignin and xylan by qRT-PCR

**Fig. 4:**
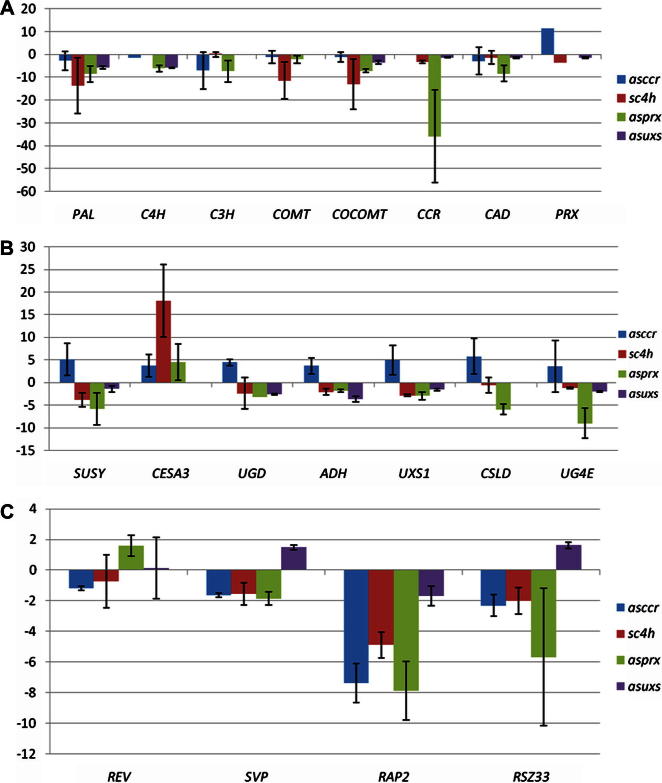
Transcriptional consequences of lignin and xylan down-regulation on (A) lignin biosynthesis (B) cell wall polysaccharide biosynthesis and (C) vascular transcription factors in lines *asccr*, *sc4h*, *asprx* and *asuxs*. qRT-PCR was performed for xylem-specific transcripts (A) lignin [*PHENYLALANINE AMMONIA LYASE* (*PAL*), *CINNAMATE 4-HYDROXYLASE* (*C4H*), *COUMAROYL-ESTER-3-HYDROXYLASE* (*C3H*), *CAFFEIC ACID O-METHYL-TRANSFERASE* (*COMT*), *CAFFEOYL-COA O-METHYLTRANSFERASE* (*COCOMT*), *CINNAMOYL-COA REDUCTASE* (*CCR*), *CINNAMYL ALCOHOL DEHYDROGENASE* (*CAD*)*]*, (B) cellulose [*CELLULOSE SYNTHASE* (*CESA*), *SUCROSE SYNTHASE* (*SUSY*)], and xylan [*UDP-GLUCOSE DEHYDROGENASE* (*UGD*), *BIFUNCTIONAL ALCOHOL/UDP-GLUCOSEDEHYDROGENASE* (*ADH*), *UDP-GLUCURONATE DECARBOXYLASE* (*UXS*), *CELLULOSE SYNTHASE-LIKE D* (*CSLD*)] and (C) xylem-specific transcripts involved in transcriptional regulation [*REVOLUTA* (*REV*), *SHORT VEGETATIVE PHASE* (*SVP*), *RELATED-TO-APATELLA 2* (*RAP2.12*), *NUCLEIC ACID BINDING/ZINC ION BINDING* (*RSZ33*)]. Fold change relative to the respective wt is shown.

Other potential major targets in xylan and lignin biosynthesis for engineering to optimise chemical and microbial digestibility for paper and biofuel manufacture are all present as their vascular specific forms, in the EST database. Primer sets were designed to profile the expression of genes associated with xylem, cortex and pith from mature tobacco plants. As a result of annotation and array analysis (Supplementary data I), genes specific for the lignin, cellulose and xylan pathways in the xylogenic cell culture were identified (Table 3). Total RNA was purified from *sc4h*, *asccr*, *asprx* and asuxs. The lignin down-regulated lines were compared to the corresponding wild type NVS while *asuxs* was compared wild type line K326 (Bindschedler et al., 2007). Internodes (1–6) showing maximum expression of secondary wall-related genes (Bindschedler et al., 2005) were profiled under an agreed protocol (see Section 4). QRT-PCR was performed on RNA samples from stems of three biological replicates from each of wild type and down-regulated lines and fold difference in expression calculated (Fig. 4).

As a general trend, all the genes of lignin biosynthesis that could be identified in the EST were down-regulated, with the exception of lignification-specific peroxidase in the *sc4h* line which was up-regulated. Our QRT-PCR results for all the genes found to be down-regulated in *asccr* are consistent with AFLP analysis performed previously in *ccr* and *cad* tobacco plants and the double transformants (Leplé et al., 2007) so this seems to be a feature of lignin down-regulated lines in tobacco. The *sc4h* showed the highest changes especially with respect to *PAL*, *COMT* and *CCOMT*. A preliminary analysis has been published for the *asprx* line based on pooled RNA samples (Kavousi et al., 2010). The present study represents an alternative rigorous measurement involving biological replicates and confirms the previous conclusions with the exception of *CCR* expression. The present study is therefore more accurate and suggests a feedback mechanism in response to lack of polymerization of monolignols in the peroxidase down-regulated line.

Lines modified for UDP-xylose provision had elevated cellulose to xylan ratios and the same or up to 20% increased lignin than wild type (Bindschedler et al., 2007). It was not possible to measure *CESA3* expression in the industrial variety K326 background as the primers were not comparable indicating varietal variation in coding sequence. This also occurred for *CSLD* and *C3H*. Expression of the lignin related genes was close to wild type levels or slightly lower, i.e. less than 2-fold, which is comparable to the levels of lignin observed (Fig. 3; Table 2). The *asuxs* line also showed general down-regulation of the genes for UDP-xylose provision including the non-target but redundant *UXS1*.

In lignin down-regulated lines, there was an upregulation of *CESA3* in all lines whereas, expression of genes involved in xylan synthesis were also upregulated in *asccr* but not in the other lines. These genes were significantly down-regulated in the *sc4h* and *asprx* lines. Alteration at the flux level clearly has different consequences than alterations at the monolignol provision and polymerization levels. However, this was not to the extent that there was any eventual compensatory increased accumulation of cellulose and xylan in the *sc4h* and *asprx* lines (Table 2). Since this preliminary data is premature to draw conclusions for the fate of xylan synthesis in *asccr* due to lack of in-depth wall composition data (Table 2). Overall there are clear indications for the existence of interdependence between the lignin and xylan pathways. It is also apparent that the levels of cellulose, xylan and lignin are not wholly dependent on transcriptional regulation of the pathways.

### 2.6 Identification of vascular specific transcription factors and profiling expression in lines down-regulated in lignin and xylan by qRT-PCR

Vascular specific transcription factors may play a role in regulation of cell wall synthesis (Vom Endt et al., 2002; Zhong and Ye, 2009). In order to identify potential novel transcription factors involved in regulation of cell wall synthesis, which may be targets for modification in biofuel crops, the seventy transcription factors (Supplementary data II) identified in the EST database were analyzed using the developmental data collection in Genevestigator (http://www.genevestigator.com/gv/index.jsp; Hruz et al., 2008). Four of these transcription factors had sequences analogous to Arabidopsis xylem specific transcription factors *REVOLUTA* (*REV*), *SHORT VEGETATIVE PHASE* (*SVP*), *RELATED*-*TO*-*APATELLA 2* (*RAP2.12*), nucleic acid binding/zinc ion binding (*RSZ33*). Expression of all these was detected in wildtype xylem tissue.

*REV* has a role in positioning and patterning of xylem tissue (Talbert et al., 1995; Emery et al., 2003; Robischon et al., 2011). Class III HD-ZIP family members, which includes *REV*, often have gene expression limited to the developing xylem. *REV* mutants have abnormal vascular structure; *rev*-10 has xylem vessels surrounding central phloem cells rather than the normal peripheral phloem surrounding the central xylem vessels (Emery et al., 2003). *REV* is upregulated in *asprx*, and its expression is suppressed in s*c4h* and *asccr* (Fig. 4). Consistently, *asprx* showed a reduction in the number of vessels (Kavousi et al., 2010) and a striking enlargement in the diameter of the surrounding fibres. This could be related to involvement of *REV* in vessel formation. *asuxs* also showed a reduction in the number of vessels. Despite no comparable increase in *REV* expression *SVP* and *RSV33* are upregulated in *asuxs*. *SVP* and *RSZ33* have not been linked to cell wall regulation before. However *RAP2.12* has a role in ethylene signalling (Lin et al., 2008) and ethylene is involved in the terminal stages of xylogenesis (Pesquet and Tuominen, 2007). Significantly, this gene was down-regulated in all lines therefore the present work indicates possible new developmental functions for these three transcription factors.

## 3 Discussion

As a result of increased interest in plant biomass as a renewable resource for industrial feedstock and especially as the basis of second generation biofuel production, there is a drive to understand the genomic basis of plant cell wall biosynthesis and modification. While the impact of the availability of and comparison of the poplar genome with the Arabidopsis and other model plant genome has been considerable, EST libraries have also significantly contributed to understanding the complexity of the genes involved in cell wall production. Thus, in an analysis of 8962 ESTs from poplar suspension cells, presumably synthesising primary cell wall (Lee et al., 2005), it was found that 62% of sequences could be fully annotated while 28% were of unknown function. The remaining 10% of EST sequences failed to show significant similarity to any proteins. These results are commensurate in classification terms with those of the ESTs used in the present study from the xylogenic tobacco cell culture described in Blee et al., 2001. However there was a clear bias towards unique sequences among unidentified clones and the EST from the tobacco line is enriched with secondary wall specific sequences. In Arabidopsis, a remarkable 7592 (28%) out of 27,139 identified functional gene sequences, are unknown, and this has prompted a search for novel genes involved in secondary wall formation by reverse genetics (Brown et al., 2005). Thus combined expression and proteomic analysis (Millar et al., 2009) also has potential for novel gene discovery and annotation in cell wall biosynthesis and modification. The major targets have been lignin and glucuronoarabinoxylan biosynthesis in relation to cellulose synthesis in the secondary wall. Co-regulation has also been studied in transgenic lines altered for lignin and xylan synthesis (Blee et al., 2001, 2003; Bindschedler et al., 2005, 2007).

The cell wall and secretory proteome from these cells has been determined by both MALDI-TOF and LC-MSMS (Millar et al., 2009). The present work describes the analysis of the gene expression patterns which give rise to the previously described proteome with respect to lignin, glucuronoarabinoxylan and cellulose biosynthesis in the secondary wall. Several approaches have been adopted to identify the genes involved in xylan synthesis in Arabidopsis, tobacco and other species. Some of these, such as *UDP-GLUCOSE DEHYDROGENASES* (*UGD*) and *UDP-GLUCURONATE DECARBOXYLASES* (*UXS*) have been cloned by homology to known candidates in other species. Functional redundancy amongst these gene families is also likely to exist in tobacco (Bindschedler et al., 2005; Molhoj et al., 2003). Proteomic approaches confirmed the identity of UXS and also led to identification of a second UGD with alcohol dehydrogenase activity. Provision of UDP-glucuronate can also take place through the myo-inositol pathway through inositol oxygenase as identified in Arabidopsis (Kanter et al., 2005). Xylan synthase itself remains to be identified. While *CSLA* codes for mannan synthase (Dhugga et al., 2004; Liepman et al., 2005; Goubet et al., 2009), *CSLE* and *CSLG* in tobacco are unlikely to be candidates simply on the basis of the expression analysis. Alternatively, a *GT43* family glycosyltransferase (Pena et al., 2007; Brown et al., 2007) or *GT47* have been suggested to be xylan synthases (Brown et al., 2009; Lee et al., 2009a; Wu et al., 2009). Added complexity may also occur through xylan remodelling. Such remodelling has been indicated already for cellulose where a role for β-glucanases in initial synthesis and assembly was ascribed (Scheible and Pauly, 2004). Similarly, the detection of xylanase in our previous cell wall proteomics study (Blee et al., 2001) and the presence of two highly similar forms in the EST collection from xylogenic cells identified in this study, may suggest a role for this enzyme in regulating xylan structure and content. These xylanases show high similarity to the Arabidopsis xylanase cDNA shown to have a role in secondary cell wall metabolism and plant development (Goujon et al., 2003). Therefore faced with such complexities, initial forays into engineering hemicelluloses have focused on the UDP-glucuronate decarboxylase step as the least likely functionally redundant step encoded for by known gene sequences. In contrast, antisense expression of the two distinct enzyme systems capable of UDP-glucose dehydrogenase activity in tobacco (Bindschedler et al., 2005) did not produce striking changes in the cell wall (Bindschedler et al., unpublished data), although over-expression of *UGD* in alfalfa did result in changes in the xylose content of wall but not the uronic acid or pectin content (Samac et al., 2004). Nevertheless *UGD* has an important role in cell wall biosynthesis (Klinghammer and Tenhaken, 2007).

Lignification has been well studied and manipulated in tobacco. Most manipulation studies of lignin have been at the various levels of flux control into phenylpropanoids and formation of monolignols, consequently focussing on the type of lignin rather than the polymerisation process. From an analysis of the accumulated data for transformations (Anterola and Lewis, 2002), it would appear that manipulation of targets at the earliest stage of lignin biosynthesis (*PAL*, *C4H*, *4CL* and *C3H*) in tobacco and *Arabidopsis*, reduces the lignin content and generally results in higher *G* to *S* ratios (Kajita et al., 1996; Sewalt et al., 1997; Blee et al., 2001a; Abdulrazzak et al., 2006), with the exception of *PAL*. This may indicate that reduction of flux through the pathway leads to selective depletion of the intermediates that go through to *S* units and *G* units, since there is growing evidence that these pathways may be differentially regulated in different cell types. However, in the peroxidase down-regulated line used in the present study there was an increase in *S* over *G* units revealed by immunocytochemistry (Kavousi et al., 2010), which must be a consequence of manipulation of the polymerisation step. This may indicate differential specificity of more than one peroxidase operating in vascular tissue and that the peroxidase down-regulated in *asprx*, *TOBACCO PEROXIDASE 60* (*TP60*) may have more activity towards guaiacyl units. As an increase in *S* over *G* units has been found for tobacco lines down-regulated for *PAL* it also indicates unpredictable variation between species and down-regulation of individual steps leads to different effects on the monomer composition of lignin. Similarly, down regulation of the later enzymes of monolignol biosynthesis, CONIFERYLALDEHYDE 5-HYDROXYLASE (F5H), CCR and CAD (Chabannes et al., 2001a,b; O’Connell et al., 2002) leads to limited effects on total lignin but with drastic and opposing changes in *S*/*G* ratios (Anterola and Lewis, 2002).

Morphologically, both *asprx* and *asuxs* lines show a reduction in vessels. One transcription factor, *REV*, known to influence vascular development (Emery et al., 2003) was upregulated in *asprx* but not *asuxs*. However, two other vascular active transcription factors, *SVP* and *RSZ33*, were up-regulated in *asuxs*. *RAP2.12*, which has a role in ethylene signalling active in the terminal stages of xylogenesis was down-regulated in all lines. With such changes it would be surprising if there were no changes to the utility of such resources.

All the lines altered for carbon flux into the phenylpropanoid pathway by down-regulation of *C4H*, monolignol type by down-regulation of *CCR* and down-regulated for a lignification-specific peroxidase that affects polymerisation, showed improved saccharification. Lines down-regulated in the pathway to xylan through antisense expression of *UXS* also showed a lower but significant improvement.

There have been comparatively fewer studies on the effect that lignin down-regulation has on other polymers and related transcription in tobacco. However the effect of *CCR* knockouts or antisense manipulation resulted in increased hemicellulose to cellulose in Arabidopsis (Ruel et al., 2009) while the opposite occurred in poplar (Leplé et al., 2007). Presented here is the composite data from our studies (Blee et al., 2001b, 2003; Bindschedler et al., 2007; Kavousi et al., 2010) as well as unpublished on the tobacco lines together with transcript profiling guided by the relevant genes found in the EST library from our xylogenic cell culture (Blee et al., 2001a). In general, most transcripts were down-regulated for both pathways in these lines while *CESA3* was upregulated in the lignin-modified lines. There are clear indications of some form of cross talk between the lignin and xylan pathways. It is also apparent that the levels of cellulose, xylan and lignin are not wholly dependent on transcription of the pathways. In addition, all the lines studied show improved enzymic saccharification. With this work it is therefore shown that regulation of cell wall biosynthesis occurs at different levels and not only at the transcriptional level providing further elements to manipulate these important pathways for industrial applicability.

## 4 Experimental

### 4.1 Plant materials

Control and transgenic suspension cultures of tobacco were derived and maintained as described by Blee et al., 2001a. *sc4h* tobacco plants were derived as described by Blee et al. (2001b) and *asprx* plants as described by Blee et al. (2003). *asccr* plants were obtained from Prof Alain Boudet, Toulouse and were as described by Piquemal et al., 1998. *asuxs* plants were as described (Bindschedler et al., 2007).

### 4.2 Growth and sampling conditions

Lines were propagated vegetatively in the greenhouse under a 16 h/8 h light-dark regime and average temperature of 25 °C. Stem material for analysis was sampled immediately prior to flowering at the 20-internode stage. Xylem tissue was isolated from designated internodes for each individual profiling parameter according to the protocol agreed by the partners in EU FPV COPOL (QLK5-2000-01493) and as outlined in previously published work (Chabannes et al., 2001a,b; O’Connell et al., 2002; Bindschedler et al., 2007; Leplé et al., 2007; Millar et al., 2009).

### 4.3 Isolation and analysis of cell walls

Acetone insoluble material of stems was isolated and fractionated as previously described (Kavousi et al., 2010). Primary walls were isolated according to Lionetti et al., 2007. The method was modified to include de-proteinisation with phenol following two days of destarching with 5U α-amylase/mg cell wall material to eliminate any remaining enzymes.

### 4.4 Enzymic saccharification

Saccharification of acetone-insoluble (cell wall) material extracted from stems of all lines was determined as described in Kavousi et al., 2010. For saccharification of primary walls extended de-starching was necessary.

### 4.5 RNA Extraction for qRT-PCR

RNA was extracted from xylem tissue taken from the fifth internode of plants at the 20th internode stage, as recommended by EU FPV COPOL (QLK5-2000-01493) and following an agreed sampling protocol (c.f. Leplé et al., 2007; Kavousi et al., 2010) using the Qiagen (USA) RNeasy minikit according to the manufacturer’s instructions.

### 4.6 Real time RT-PCR mRNA quantification

DNAse-digested total RNA was reverse transcribed to generate cDNA using the QuantiTect® Reverse Transcription kit (Qiagen, USA). The selected tobacco cDNAs (Table 3) were amplified using the Quantace Sensimix NoRef SYBR green master mix (Quantace, UK) to determine real time mRNA quantification. Primers were designed spanning intron exon boundaries where possible from tobacco EST and cDNA sequences using the Primer3 oligonucleotide design web tool (Rozen and Skaletshy, 2000). Where these were inefficient, the EST was used to identify the corresponding full-length clones in Genbank and these were used to design satisfactory primers. The primers used in this study are listed in Table 1. The relative expression levels of each gene were interpolated from standard curves generated from serial dilutions of cloned fragments of the genes of interest. 18sRNA and ElF4 were used as house-keeping reference genes. The differences in expression profiles of the genes of interest were calculated by normalization to 18sRNA. Standard amplification protocols were used and carried out on the Corbett Research RG-6000 real-time PCR machine (Corbett Research, Australia). Reaction volumes of 20 μl were used for each run, which were performed in triplicate. Relative levels of transcripts were determined using established methods (Pfaffl, 2001).

## Figures and Tables

**Table 1:** ESTs identified in an EST collection from a xylogenic tobacco cell culture (Blee et al., 2001b) involved in synthesis or modification of cell wall components.

Function	Number of genes	Function	Number of genes
*Lignification*		*Polysaccharide biosynthesis*	
Cinnamic acid 4-hydroxylase (C4H)	3	UDP-glucuronate decarboxylase (UXS)	4
Cinnamoyl CoA reductase (CCR)	3	ADH-like UDP-glucose dehydrogenase (ADH)	10
Cinnamyl alcohol dehydrogenase (CAD)	7	UDP-glucose 6-dehydrogenase (UGD)	2
Catechol O-methyltransferase (COMT)	7	UDP-glucuronate 4-epimerase (UG4E)	1
Caffeoyl CoA O-methyltransferase (CCOMT)	4	Cellulose synthase-like (CSL)	2
Peroxidase	20	Glucosyltranferase	2
*Cell wall modifying proteins*		Glycosyltransferase	8
Pectinesterase	9	Xylosyl transferase	1
Extensin	3	Sucrose synthase (SUSY)	5
Proline-rich protein	5	Invertase	3
Glycine-rich protein	5	ADP-glucose pyrophosphorylase	9
Expansin	4	Cellulose synthase (CESA)	2
Pectin methylesterase	4	*Transcription factors*	
Xyloglucan enotransglycosylase	6	Total	70

**Table 2:** Cell wall composition of WT tobacco and transgenic lines. (A) Polysaccharide composition of leaf primary cell wall and stem secondary cell wall as biomass analysed for saccharification. Data from the most recent fractionation is given with standard deviation, and previously published data is given in brackets. (B) Detailed analysis of secondary cell wall.

Line	Line (abbreviation)	Leaf primary wall composition (%)			Stem secondary wall composition (%)		
		Pectin	Hemicellulose	Cellulose	Pectin	Hemicellulose	Lignocellulose
(A)							
WT (*Nicotiana tabacum* v. Samsun)	WT (NVS)	25 ± 2	17 ± 2	40 ± 1	<4	22 ± 4 (23)	63 ± 4 (67)
Sense cinnamate-4-hydroxylase^a^	*sc4h*	26 ± 3	17 ± 2	40 ± 1	<4	25 ± 2 (23)	57 ± 2 (58)
Antisense cinnamoyl Co-A reductase^b^	*asccr*	28 ± 2	15 ± 1	43 ± 3	<4	22 ± 2	68 ± 4
Antisense tobacco peroxidase 60^c^	*asprx*	28 ± 3	16 ± 1	41 ± 2	<4	24 ± 1 (23)	60 ± 1 (63)
WT (*Nicotiana tabacum* K326)	WT (K326)	25 ± 2	17 ± 1	39 ± 2	<4	20 ± 2 (23)	55 ± 1 (69)
UDP-glucuronate decarboxylase^d^	*asuxs*	26 ± 3	15 ± 3	42 ± 3	<4	10 ± 1 (18)	63 ± 3 (73)

Line	Line (abbreviation)	Lignin content (%)	*S*/*G*	Glucose content (%)	Xylose content (%)	Glucose/xylose	Secondary wall phenotype (%)

(B)							
WT (*Nicotiana tabacum* v. Samsun)	WT (NVS)	20.15 ± 0.12	0.82	46.7	22.8	2.05	Normal
Sense cinnamate-4-hydroxylase^a^	*sc4h*	14.67 ± 0.17	0.83	43.0	23.05	1.86	Thickened fibre walls; vessels/tracheids normal
Antisense cinnamoyl Co-A reductase^b^	*asccr*	15.4 ± 0.9	1.64	n.d.	n.d.	n.d.	Diffuse S2 layer
Antisense tobacco peroxidase 60^c^	*asprx*	15.42 ± 1.18	1.21	47.7	22.6	2.1	Diffuse S2 layer
WT (*Nicotiana tabacum* K326)	WT (K326)	20.2 ± 0.2	n.d.	49.03	22.97	2.23	Normal
Antisense UDP-glucuronate decarboxylase^d^	*asuxs*	24.5 ± 0.5	n.d.	49.12	17.98	2.72	Zonation in S2

G=guaiacyl, S=syringyl.

a Blee et al. (2003), polysaccharide data not previously published.

b O’Connell et al. (2002).

c Blee et al. (2003) and Kavousi et al. (2010).

d Bindschedler et al. (2007).

**Table 3:** Primers used for qRT-PCR.

Gene description as per NCBI	Abbreviation	Accession Nos.	Forward (5′ to 3′)	Reverse (3′ to 5′)	Amplicon size (bp)
*Cell wall synthesis*					
*PHENYLALANINE AMMONIA LYASE*	*PAL*	D17467	GCAAACAGCTCAATCTTCCA	TCGACTTCTTTTGGCAACAC	74
*SECRETORY PEROXIDASE*	*PRX*	AF149251	CTTGCCAACAAGCTCCACTA	CAAAGGAAGGGGAAAAGTGA	76
*CINNAMOYL CO-A REDUCTASE*	*CCR*	AY149609	TGTGTCTTCTGTTGCTGCTG	ATTCACTGTCCGACCAACAA	83
*CINNAMYL ALCOHOL DEHYDROGENASE*	*CAD*	EH664196	TGGAACATCTTGGTGCAGAT	ATGGCCAACAGGGACAGTAT	107
*CATECHOL O-METHYLTRANSFERASE*	*COMT*	EH663855	ACATAACCCAGGAGGCAAAG	TTCCATGACCCAAGTGTTGT	114
*CAFFEOYL CO-A METHYL O-TRANSFERASE*	*COCOMT*	EH665253	ATTTTCGTGGATGCTGACAA	GTCGTAGCCAATCACACCAC	90
*CINNAMATE-4-HYDROXYLASE*	*C4H*	EH664914	AGCAATGCTCTGAAATGTGC	CCTCAGTTGATCTCCCCTTC	67
*P-COUMARATE-3-HYDROXYLASE*	*C3H*	EH663728	AGCAGTGGCCTTTAACAACA	GTCACCATCACACTTCAAAGG	75
*SUCROSE SYNTHASE*	*SUSY*	EH664745	GAAGCAAGGACACTGTTGGA	ATACAATCCAGGCATCGTGA	62
*CELLULOSE SYNTHASE 3A*	*CESA3*	EH663724	TGGAATTGATGAATGGTGGA	CAACCCTTGGAAGACCTAGC	90
*UDP-GLUCURONATE DECARBOXYLASE*	*UXS*	EH663981	AAAACCACCACCAGAACCAT	CAATAAATCCAGCACCACCA	93
*CELLULOSE SYNTHASE LIKE D*	*CSLD*	EH665280	GGAAAGGAACTTGGAAGTGG	AATCTGCACAATCCCACGTA	80
*UDP-D-GLUCURONATE 4-EPIMERASE*	*UG4E*	EH664843	GGGGTCGTATTTGTGTTTCC	TGTTTCTCCCAATGATGACC	88
*UDP-GLUCOSE DEHYDROGENASE*	*UGD*	EH663670	AATGAGTCCAACAACCGTGA	TCCTTTGTTGCTGTGTAGGC	63
*ADH-LIKE UDP-GLUCOSE DEHYDROGENASE*	*ADH*	AY619949	AATGCCATGTCAGCTCTTTG	AATGCCATGTCAGCTCTTTG	60

*Transcription factors with xylem specific expression*					
*RELATED TO APETALA2*	*RAP2*	EH665541	CGAGGTGTGAAGGTTGAGAA	CCACGGTCTCTGCCTTATTC	82
*SHORT VEGETATIVE PHASE*	*SVP*	EH665729	CCACGGTCTCTGCCTTATTC	GGTCAATCCAGCTTCCAGAG	85
*ARGININE/SERINE RICH ZINC KNUCKLE CONTAINING PROTEIN*	*RSZ33*	EH663821	TGGAGGACGTCTTTAGCAGA	CATCAGCATCTCGAGGATCA	98
*REVOLUTA*	*REV*	EH663642	GCTGTCGATATGCAGAGGAA	CAGCAGTTCCTGTAGCCTTG	62
