# Supplementary material for: Transcriptional changes related to secondary wall formation in xylem of transgenic lines of tobacco altered for lignin or xylan content which show improved saccharification
Source: Phytochemistry. 2012 Feb;74:79–89. doi: 10.1016/j.phytochem.2011.10.009 (PMC3657182; doi:10.1016/j.phytochem.2011.10.009)
Supplement: Supplementary data 3 [file mmc3.doc]

##### Supplementary Methods I: EST library construction, sequencing and annotation

#### *Total RNA extractions and poly-A^+^ mRNA purification*

RNA for the cloning of the original EST library was extracted from tobacco plants cultivar NVS, AsC4H, AsPRX or AsCCR and from tobacco cell cultures with guanidium thiocyanate-phenol according to Chomczynsky and Sacchi (1987) with slight modifications. Insoluble material was removed from the homogenisation buffer by centrifugation at 1000 g for 15 min prior to addition of chloroform. After the first precipitation with isopropanol, the RNA pellets were washed with 75% ethanol and directly dissolved in 0.5% SDS. Solubilisation was facilitated by incubation at 55^o^C followed by centrifugation in a microfuge for 10 min at 10 000 rpm for removing insoluble particles. Three day-old xylogenic tobacco cell cultures were used to construct a cDNA library. PolyA+ RNAs were isolated from total RNAs with a PolyAtract II kit (Promega). cDNAs were synthesised, selected by size, ligated into λZAP II UniZAP-XR vector (Stratagene) and packaged into Gigapack Gold III packaging extract (Stratagene). Isolated cDNAs were excised in vivo from λZAP II for further analysis in pBluescript SK phagemid (Stratagene).

*Sequencing of cDNA inserts*

Following positive quality assessment, 3000 colonies from the EST library were picked into 96-well microtitre plates containing 100 µL aliquots of LB medium containing 100 µg/mL ampicillin. Following cell growth at 37 °C, sterile glycerol (final concentration) 25%was added to each well and the plates stored at −85 °C. To assess library quality and redundancy, 96 cDNA clones were sequenced for each library and sequence analysis (database mining) performed. Sequencing templates were prepared via colony polymerase chain reaction (PCR) (Saiki et al., 1988) in a 96-well format. Following PCR amplification of the template, a dye terminator cycle sequencing protocol was followed (as described for the MegaBACE 1000 capillary sequencer (Amersham Biosciences)). Sequence clean-up and blast searching were performed using a custom laboratory information management (LIM) system as described in Wilson et al., 2004. A total of 2976 clones were subject to single pass sequencing from the 5’ and the resulting 2688 expressed sequence tag (EST) sequences can be downloaded from GenBank public (EH663598 to EH666265). Sequences were subjected to a search of a nonredundant database comprising all non-redundant GenBank CDS translations + RefSeq Proteins + PDB + SwissProt + PIR + PRF using the blastx search algorithm.

*Microarray generation, hybridizations and data analysis*

Templates for printing microarrays were produced via colony PCR in the presence of 5’- amino-C6-labelled T3 and T7 primers (Stratagene). PCR products were ethanol precipitated and resuspended in 1xGSSA spotting solution (Genetix Ltd) prior to printing. Templates were transferred to 384-well microtitre plates in a dispersed manner to ensure that clones from each library were represented in every subarray. This dispersal did not substantially split clones from the same 96-well plate, allowing clones to be tracked by plate number and well identifier in the original library glycerol stocks. A total of 2688 clones from the cDNA library produced were spotted in duplicate onto Amersham CodeLink activated glass slides, alongside several internal controls. Amplified inserts of plasmids coding for PAL, xylanase, UDP-glucuronate decarboxylase were also spotted for use as potential positive and negative controls, together with PCR products containing Poly-dA_(50)_ and the pBlueScript II SK+ multiple cloning site. The array format was 48 subarrays each containing 288 spots (a 17 × 17 layout with the last position left blank). Control clones were arranged such that copies of each control were equally dispersed across the array. PCR products were spotted onto CodeLink activated microarray slides (Amersham) using a BioRobotics Microgrid II arrayer (Genomic Solutions Ltd). The exact array format and positioning of individual clones may be viewed as an Excel spreadsheet contained in the supplementary material. Immediately after spotting, slides were placed at 21 °C in an airtight container above a saturated NaCl solution for 24 h before storage in a dust-free, dessicated environment before use.

## **Microarray hybridisation and normalisation strategies**

Microarray slides were hybridized in reverse dye- labelling experiments using Amersham Cy3/Cy5-dCTP dye (Amersham) conjugated cDNA probes. The probes were generated from pooled total RNA samples for xylem, cortex and pith from mature tobacco plants (cultivar NVS) and the Tcyt transformed tobacco cell culture line, as well as an untransformed control tobacco cell culture line. cDNA derived from three separate RNA extractions for each of the cell and plant lines were hybridised to the microarray. Total RNA samples (50 µg) were reverse transcribed in the presence of Cy3 or Cy5 dCTP to produce labelled first-strand cDNA which was purified using QIAGEN MinElute PCR purification spin columns. The eluted cDNA samples were then used immediately in array hybridization experiments. Two colour competitive hybridisations were performed as previously described by Stewart et al. (2002), hybridising the test RNA-derived cDNA against the EST cDNA from the 3 day-old transgenic cell culture. The hybridised slides were scanned sequentially at 532 and 635 nm corresponding to Cy3 and Cy5 excitation maxima using the 4.28™ Array Scanner (Affymetrix). Comparative spot intensities from the images were calculated using Imagene 4.0 (BioDiscovery), and imported into GeneSpring 4.2 (Silicon Genetics) for further analysis. The array data was normalised to the 50^th^ percentile, and values of less than zero were adjusted to zero. Repeat hybridisations using the same cDNA samples (between 3 and 7 replicates for each condition) were replicated together. The experiments were then normalised to the untreated control sample using a per gene normalisation strategy.

*Microarray data analysis*

Two measures of significance were applied to the normalised data set to identify differentially regulated genes: (i) a minimum *p*-value of 0.05 incorporating the cross-gene error model (GeneSpring) was set. This discriminated genes significantly deviating from the 1:1 ratio (treated : untreated). The genes were then subjected to Benjamini and Hochberg correction to take into account multiple experiment testing; and (ii) a one-way ANOVA (GeneSpring).

A technique of single spot replacement (SSR) (K. Laing, personal communication) was also used to enhance the original data set. The un-normalised cDNA : genomic DNA ratios for each replicate under each condition were imported into Microsoft Excel. For each element on the microarray the individual ratio furthest from the median of the replicates was replaced with the mean of the remaining ratios. In this way the effect of extreme values was minimised from the data set. This SSR data set was then normalised as previously described, and subjected to two measures of significance: (i) the statistical group comparison (ANOVA); and (ii) identification of genes which were differentially expressed in the normalised data sets using the statistical package SAM (Significance Analysis of Microarrays) version 1.15 (Tusher et al., 2001). The minimum fold change between control and drug-treated data sets was 1.5, and a false discovery rate (FDR) of less than one (of the median) was used as a measure of significance.

EST library characterization

EST sequences from the EST library (Genbank EH663598 – EH666265) were inputted into NCBI BLAST search (<http://blast.ncbi.nlm.nih.gov/Blast.cgi>). ESTs were annotated with the first significant (p = <0.05) BLASTN (nucleotide blast) hit describing a gene in the top 50 hits. Genbank entries which were not described as a gene but were labelled as e.g. ‘chromosome clone’ were not included unless there was no alternative.

Chonczynsky, P., Sacchi, N. 1987. Single step method of RNA isolation by acid guanidium thiocyanate –phenol chloroform extraction. Anal. Biochem. 162, 156-159

Dudoit, S., Shaffer, J.P., Boldrick, J.C. 2003 Multiple hypothesis testing in microarray experiments Statistical Science 18,71-103.

Saiki, R.K., Gelfand, D.H., Stoffel, S., Scharf, S.J., Higuchi, R., Horn, G.T., Mullis, K.B., Erlich, H.A. 1988 Primer-directed enzymatic amplification of DNA with a thermostable DNA polymerase. Science 239, 487–491.

Tusher, V.G., Tibshirani, R., Chu, G. 2001 Significance analysis of microarrays applied to the ionizing radiation response. Proc. Natl. Acad. Sci. 98, 116–5121

**Legends to Supplementary data files**

**Supplementary data I:** EST library constructed from xylogenic tobacco cell culture Tcyt. Methods of library construction and annotation are described in Supplementary Methods I. Column A shows the results of microarray analysis and genes that differ in expression by more than twofold between the xylogenic tobacco cell culture and the wildtype culture. Red = up-regulation, Green = down-regulation

**Supplementary data II:** Transcription factors identified and extracted from Suuplementary data I
